# Adjuvant Chemoradiation Therapy in Gastric Cancer: Critically Reviewing the Past and Visualizing the Next Step Forward

**DOI:** 10.1155/2015/650846

**Published:** 2015-05-26

**Authors:** Konstantinos Papadimitriou, Georgios Antoniou, Christian Rolfo, Antonio Russo, Giuseppe Bronte, Vassilios Vassiliou, Demetris Papamichael, Marc Peeters, Panteleimon Kountourakis

**Affiliations:** ^1^Department of Medical Oncology, University of Antwerp, 2650 Antwerp, Belgium; ^2^Department of Medical Oncology, Royal Marsden Hospital NHS Foundation Trust, London SW3 6JJ, UK; ^3^Department of Surgical and Oncology Sciences, University of Palermo, 90127 Palermo, Italy; ^4^Department of Radiation Oncology, B.O.C. Oncology Centre, 2006 Nicosia, Cyprus; ^5^Department of Medical Oncology, B.O.C. Oncology Centre, 2006 Nicosia, Cyprus

## Abstract

Gastric cancer remains one of the most common malignancies worldwide. Despite the significant advances in surgical treatment and multimodality strategies, prognosis has modestly improved over the last two decades. Locoregional relapse remains one of the main issues and the combined chemoradiation treatment seems to be one of the preferred approaches. However, more than ten years after the hallmark INT-0116 trial, minimal progress has been made both in terms of effectiveness and toxicity. Moreover, new regimens added to combined therapy failed to prove favourable results. Herein, we attempt a thorough literature review comparing pros and cons of all relative studies and potential bias, targeting well-designed future approaches.

## 1. Introduction

Gastric cancer (GC) remains one of the most common types of cancer worldwide accounting for about 9.9% of new cases [1, 2]. Despite the significant advances in surgical treatment and multimodality strategies, prognosis has only been modestly improved over the last two decades. In fact more than 50% of patients (pts) diagnosed with GC in 2008 in the United States are expected to die [3]. Appropriate resection and early diagnosis remain the key points for a successful approach in GC treatment [4, 5]. Until the late 90s surgical resection alone was the most commonly used treatment even for high risk resectable GC. However, most pts are not cured by surgery alone and the high rate of relapses suggests the need for additional treatment. Multimodality strategies incorporating adjuvant chemotherapy (CT), perioperative chemotherapy, and neoadjuvant and adjuvant chemoradiation (CRT) have been tested in clinical trials. In an attempt to understand the role of adjuvant CRT in resected GC, but also the less than expected progress done in this field the last decade, we attempted a thorough review. Possible factors of discrepancy and bias of the most important trials are presented and to date the optimal treatment strategy is yet to be defined.

## 2. Multimodality Options

In an attempt to improve outcomes for pts with locally advanced resectable GC researchers studied different combinations of adjuvant and neoadjuvant CT regimens with or without RT. Multiple prospective randomized studies addressed the question of the best multimodality approach for these patients. Incorporation of neoadjuvant and adjuvant CT, as well as adjuvant CRT over surgery alone, resulted often in conflicting results.

In fact, early trials for adjuvant CT proved only a modest benefit in survival rates compared with surgery alone, while subsequent trials failed to demonstrate consistent benefit for systemic adjuvant CT [6–9]. Meta-analyses and recently published trials, mostly coming from Asia, presented a positive impact for adjuvant CT [10]. Between them the CLASSIC and the ACTS-GC phase III randomized trials clearly demonstrated that adjuvant CT, in the form of XELOX and S-1 respectively, confers significant survival advantage in pts following margin negative D2 gastrectomy [11, 12]. In the meta-analyses, including 31 eligible trials, results also favored adjuvant CT, but differences in surgical techniques and multiple design weaknesses have been noted [10]. Still, these results are not replicated in the western hemisphere where surgical techniques are different, while the use of S-1 is not commonly selected. For these reasons adjuvant CT is not the standard of care for resected GC in western countries. In addition, CT alone in the neoadjuvant setting failed also to provide consistent results [13–15]. On the other hand, the well-designed MAGIC trial and other similar trials established perioperative approach as the standard of care in Europe [4, 14, 16]. In USA, adjuvant CRT is the preferred option due to the results of the INT-0116 trial [17]. These two approaches are considered as standard of care in western countries, but there remains no consensus over the best approach to treatment.

The reasons for this luck of uniform treatment strategy selection include variation in surgical techniques and selected regimens, as well as in patient population [18–20], but are mostly related to the small body of available studies comparing directly, in a face to face design, the different multimodality strategies. Among these, 5 Asian trials evaluated adjuvant CRT versus adjuvant CT [21–25], but only one trial was able to individually show a benefit to adjuvant CRT over adjuvant CT [21]. In the latest published trial in this setting, the ARTIST trial, the addition of RT to CT when compared to CT only conferred a significant reduction in the primary outcome of recurrence but only in pts with nodal involvement [25]. Therefore, the ongoing ARTIST-II trial will compare adjuvant CT with or without RT for completely resected GC with nodal involvement. A meta-analysis including all five Asian trials resulted in a positive association between improved survival and RT addition [26]. Still, a detailed comparison of the different multimodality options in the treatment of GC will not be further analyzed in this paper since our aim is to focus on CRT and validate the status and its progress. In Table 1, major trials for curative strategy in GC are shortly presented.

## 3. CRT Rationale and the Early Trials 

The locoregional recurrence is one of the early concerns in GC. In fact its incidence is 40–80%, after resection with curative intent [27, 28]. The high frequency of such relapses suggests the need for the addition of a local treatment approach to systemic therapy. For that purpose the combination of RT with CT could be an attractive strategy. Indeed, it was since the early 80s when the first phase III trials evaluated the role of CRT in resected GC [29, 30]. The importance for local disease control was also evident from early phase III trials on pts with residual or unresectable disease. Approximately 20% of that population were long-term survivals when treated with CRT [31, 32].

Different subsequent trials validated a variety of CRT combinations, in the neoadjuvant, adjuvant, and perioperative setting [33–35]. The survival benefit from combined modality therapy has become clearer over time, but there is no consensus which is the optimal approach. During the last decade, adjuvant CRT has become one of the most common treatment strategies for GC pts after curative resection in the US. The small phase III trial of the Mayo clinic was one of the first trials evaluating positively the results of a trimodality treatment, with surgical resection followed by CRT [29]. Those early favorable results were verified in the early 2000 by the INT-0116 trial which is considered the hallmark in adjuvant CRT [17]. This trial changed the standard of care in the US for high risk pts with GC. Despite its promising results, over 50% of pts still died within 3 years from surgery. Furthermore in this trial, 17% pts did not complete the planned CRT protocol due to treatment related intolerance. An updated report of the INT-0116 with a median follow-up of 10 years confirmed the earlier results, with OS and RFS continuing to demonstrate dramatic benefit for patients who received adjuvant CRT [36]. The use of infusional instead of bolus 5FU is a point of discussion, as many clinicians prefer this form of administration extrapolated from the practice from rectal cancer. Still no randomized studies compared infusional versus bolus 5FU in CRT for GC. Preliminary data suggest that the approach of infusional 5FU could be an acceptable alternative, with favorable toxicity and tolerability [37].

The INT-0116 phase III study included the combination of RT with bolus administration of 5FU and leucovorin (LV), with clear benefit in overall survival (OS) and disease-free survival (DFS), over surgery alone. Median DFS was 30 in CRT group versus 19 months in surgery only group (*p* = 0.0002); median OS was 40 versus 26 months. A statistically significant improvement in 3-year DFS of 48% was reported; 3-year OS rates were 50%, when in surgery only group were 41%. Though stage IB–IV pts were included, still the effectiveness in T2N0 was not clear. Also after D2 lymphadenectomy the role of adjuvant CRT is unclear because only 36% had D2 in the study. Recently, long-term results from the extended INT-0116 with a 10-year follow-up confirmed a persistent improvement in OS and DFS rates in pts treated with CRT, in all disease stages included in the trial [36, 38].

## 4. The “Followers” of INT-0116: Detailed Result Analysis and Comparison

A large observational Korean study, that followed one year later the INT-0116, confirmed the positive outcomes. Patients after D2 resection were treated with a similar CRT regimen. A very high percentage of RFS (75.5 months) and an impressive 5-year OS of 57% were reported [39]. Median OS and DFS were 95.3 and 75.6 months in the CRT group versus 62.6 and 52.7 months, respectively, in the D2 resection group. Although these results are not derived from a randomized controlled trial and are subject to several biases, they imply a potential positive role of CRT even in pts with D2 resection and provide indirect support for the CRT benefit in adequately surgically staged pts.

Additionally, at least two meta-analyses of randomized trials incorporating CRT in the adjuvant setting demonstrated a survival benefit with the addition of RT [40, 41]. The first meta-analysis included 9 eligible randomised controlled trials, 4 of preoperative RT (832 pts) and 5 of postoperative CRT (869 pts); adjuvant CRT significant reduced the 5-year (OR 0.45; 95% CI 0.32–0.64; *p* < 0.00001) mortality rate compared to surgery alone; still authors concluded that available evidence is inadequate to determine whether postoperative CRT is superior to preoperative RT. The second meta-analysis included 9 randomized clinical trials (2,025 pts) in which RT (preoperative, postoperative, and/or intraoperative) was compared with surgery alone or surgery plus chemotherapy. Of them 5 trials were classified as high quality and 4 as low quality. The addition of RT resulted in significant 5-year survival benefit; using an intent to treat (ITT) and a Per Protocol (PP) analysis, the overall 5-year RR was 1.26 (95% CI: 1.08–1.48; NNT=17) and 1.31 (95% CI: 1.04–1.66; NNT=13), respectively. Though, the statistically significant improved likelihood of 5-year survival was limited in the subgroups of lower quality trials, trials with a LQED_2_ under 40 Gy, trials using preoperative RT, trials not using intraoperative RT, and trials published after 1990. On the other hand, a late Dutch observational study enrolled 694 pts who underwent D1 or D2 surgery. Ninety-one pts underwent postoperative 5FU-based CRT, five pts with 5FU/leucovorin, 39 pts with capecitabine, and 47 with capecitabine and cisplatin. Additional benefit was observed after a D1 dissection. On the contrary, no improvement was revealed in outcomes in patients had a D2 surgery. In R1 resection, postoperative CRT was significantly associated with better survival [42].

Furthermore, in a population-based analysis, data from the Oregon State Cancer Registry were analyzed to assess the treatment approach of GC before and after 2001, when INT-0116 was published [43]. Survival outcomes, in the Oregon cohort, for pts undergoing CRT were 20 months, when the INT116 reported 36 months. In pts in the surgery only arm, the median survivals were 15 versus 27 months for Oregon and INT116 cases, respectively. In this study a modest survival benefit was associated with CRT, but this benefit did not reach statistical significance. Several causes could be related with these results compared to similar cases of the INT116. In fact, pts in a clinical trial always do better as compared to that of a population-based cohort. Patients enrolled in any clinical trial comprise a “special” subset of all cases resulting in a possible selectivity bias. Age was also an important factor, with the median age of CRT pts being substantially younger than the median age of those not receiving CRT (64 versus 75 years). Interestingly the median age in INT-0116 was 62 years. It is indeed likely that older pts have more comorbidity that would interfere with tolerance to CRT. The subset analysis of the extended INT-0116 strongly suggests that cases with poorly differentiated diffuse histological type did not benefit from CRT, whereas cases with intestinal-type histology did benefit [38]. This is important when comparing the two trials, since 22% of the Oregon registry cases had signet ring, poorly differentiated pathology that was not expected to have additional benefit from CRT.

In addition, the effect of differences in design and selection criteria between trials is further extended in reported toxicity profiles. For example, in the INT-0116 significant toxicity was reported despite the high standards in pts' enrolment. Overall grade ≥3 toxicities occurred in 73% among 273 pts with available toxicity information, including high rates of overall hematologic and gastrointestinal toxicity in 54% and 33%, respectively. Grade III and IV toxicities were reported in 41% and 32%, respectively. Gastrointestinal grades III and IV toxicity rates were 29% and 3%, respectively, whereas grades III and IV hematologic toxicities were observed in 26% and 28% of pts [44]. Furthermore, only 63% of the pts were able to complete treatment as planned. Interestingly in the Korean study, despite the extent of the surgical procedure the toxicity profile was much better than that of the INT-0116, with toxic effects (grade ≥ 3) cumulatively reported in 40% of the pts. Grades III and IV GI toxicity rates were 14.3% and 0.6%, respectively, whereas grades III and IV hematologic toxicities were observed in 23.5% and 6.4% of pts. 75.2% of the pts completed the treatment protocol. It should be highlighted that the two trials had essentially followed the same CRT protocol, with only a slight difference in total doses of 5FU (8.800 mg/m^2^ in Korean trial versus 9.175 mg/m^2^ in INT-0116) but this alone could not explain the important difference in toxicity profile. The differences are more likely related to selection criteria.

Furthermore, multilinguality could be another possible reason for discrepancy in results. D'Angelica et al. underline that there is a high variability in relapse patterns in GC in the literature [45]. This variability is multifactorial and relates to inconsistencies in treatment and differences in tumor biology, as well as in the mode and timing of detection of relapse. Also, discrepancies regarding the definition of relapse terms like local, regional, locoregional, and distant also correlate with the diversity in relapse profile. In addition, minor differences in RT techniques and fields may also interfere with the results.

## 5. The Role of Surgical Procedure and Staging

Differences in surgical staging procedures when comparing trials have also been the subject of extensive debates. Indeed, it is important to note that the results are highly amended by the completeness of the surgical procedure, parameter further emphasized by the striking results of the Korean trial and also the differences in overall TNM stage stratification. In fact in the Korean study, 98.2% of the pts had more than 15 lymph nodes (LNs) and 86.9% had more than 25 LNs removed; 55.9% had total gastrectomy. Furthermore, a relatively early TNM stage distribution was noted, with more than 65% of the study population classified as stage II and IIIA (AJCC). In the INT-0116 trial, although no comparable data are published, the less radical surgical procedures and the overall more advanced stage, as defined by the high number of less than D1 resections (54%) and the more advanced distribution of T and N category, may interfere with the poorer results, when compared with that of the Korean trial. In the INT-0116, although D2 dissection was recommended, it was performed in only 10%, while 54% did not even have D1 resection. This limited extent of the surgery is probably related to the inferior survival and the high relapse rate (64%) in the “surgery only” arm and became the main cause of criticism for the INT-0116. Of notice is that in the Oregon observational study even pts with R1 resections were included and about 70% of them had less than 15 nodes examined, making the staging data in these cases suspect.

Long-term survival after adequate surgical treatment without CT or CRT has also been reported in different studies [46, 47]. Controversially, other trials question the role of extensive surgical approaches in survival and relapse in GC [48]. Despite these discrepancies a minimum of removed LNs and a clear margin are mandatory, according to international treatment guidelines. A number of early prospective trials failed to prove a survival advantage for more extensive gastric resections or for more extensive lymphadenectomy [49–55]. The high rate of regional node relapse may provide a partial explanation for the lack of survival benefit with more extended dissections in these surgical trials implying an early systemic nature of the disease. Probably, multicenter trials and the involvement of many different surgeons especially from small volume centres should not be considered ideal, when evaluating surgical results. On the other hand, the recent Dutch D1/D2 trial correlates the D2 approach with lower locoregional rates of recurrence despite the higher perioperative mortality [56]. Subjectiveness of surgical procedures and differences of surgical techniques between early and late trials may in part explain this discrepancy.

Since the mid 90s Bunt et al. convincingly demonstrated that adequate staging requires a full assessment of N1 and N2 LNs [57]. In this study, the pts undergoing D2 gastrectomy were assigned a tumor stage by initially evaluating only the N1 nodes. Subsequently, the N2 nodes were examined and the final stage was assigned. The evaluation of N2 nodes increased the overall stage. For example, 60%–75% of cases with stage-III disease were upgraded to stage IIIB or IV. These results confirm that examining a large number of LNs is mandatory for adequate staging. With this in mind both Oregon and INT-0116 pts may be understaged due to poor assessment of LNs metastases and therefore the addition of adjuvant treatment may have proved beneficial. A modified D2 lymphadenectomy, with pancreas and spleen preservation, is now considered an appropriate surgical approach for experienced surgeons who can perform the procedure without increased surgical morbidity or mortality. Extending dissection beyond D2 to para-aortic nodes has not shown survival improvement.

## 6. Newer Regimens and Strategies

During the last decade different CT regimens combined with RT have also failed to robustly prove a substantial additional advantage in DFS or OS, while significant toxicity and tolerability issues were always present. In fact, in the RTOG-0114 trial, the incorporation of Paclitaxel–Cisplatin (P) containing regimens to CRT failed to prove additional advantage. It should be noted that the unacceptable high rate of GI toxicity leads to discontinuation of randomization. The participating institutions were notified to inform pts of the increased toxicity of regimen and additional treatment was to be given at the discretion of their treating physician [58]. On the other hand, in the early phase II study of Kollmannsberger et al., which evaluated the addition of paclitaxel to adjuvant CRT using 5FU/LV/P, toxicity was acceptable in both arms. Projected 2-year PFS was 61% and 64% in the two groups, respectively, but the rate of early treatment discontinuation was 26% [59]. In a small Asian trial, comparing CRT with a 5FU/P based regimen versus CT only, no advantage of the CRT approach was proven but, instead, severe toxicity was also reported [60]. In another small phase I early trial P seems promising when incorporated in CRT with infusional 5FU/LV, in contrast to the results of the previous mentioned studies, but efficacy has yet to be established [61].

Even without RT combination CT regimens are not easily tolerated in this group of pts. In the FFCD 8801 phase III trial, adjuvant 5FU/P also caused tolerability issues due to toxicity; only half of them received more than 80% of the cumulative planned dose of CT [62]. However, capecitabine- (X-) based CRT has been shown to be well tolerated although effectiveness has to be proven. This approach is not as common in western countries compared to Asia [63, 64]. These data indicate that toxicity and tolerability are a major issue in this patient population, influencing the results of effectiveness independently. Moreover, the discrepancies between these data, probably related to the small number of pts and the early study design in most of these trials, do not allow conclusive results.

Even the recent, randomized CALGB 80101 trial, that incorporated the Epirubicin-Cisplatin-5FU (ECF) in a “sandwich” regimen to CRT with 5FU/LV, failed to prove an advantage versus the classic INT-0116 regimen [65]. OS and DFS were comparable with the results of the INT-0116, which was conducted more than 10 years ago. However, definitive conclusions on the benefit of a more intensified CT regimen from this trial cannot be drawn since a low dose intensity of the ECF was selected. Interestingly, the researchers used infusional instead of bolus 5FU as in the original regimen of the INT-0116, when given concurrently with RT in both arms but 5FU/LV was given bolus before and after RT in the no ECF arm. This unexpected modification discourages a comparison with the investigational arm protocol in terms of toxicity and tolerability. Additionally, control data for surgical approach selected in this trial are not published, parameter that was subjected to major criticism in the Intergroup 0116 trial.

In a different setting the phase III trial of Stahl et al. validated the role of CRT prior to surgery compared to CT only. Preliminary results point to a survival advantage for preoperative CRT compared with preoperative CT, but unfortunately the study was prematurely closed due to low accrual [66]. Limited experience coming from single institute or retrospective trials also implies a potential role of CRT in neoadjuvant setting, but mature data in this field remain poor [67–69].

## 7. Late and Ongoing Trials

The same investigators that published the early retrospective Korean trial recently reassessed the role of CRT in resected GC, for the first time, in a large scale prospective phase III design (ARTIST trial) [70]. Six cycles of XP were compared with two cycles of XP followed by RT with X and then followed by two additional cycles of XP (XP/XRT/XP). Overall, the addition of CRT in pts with D2 resection did not significantly prolong DFS (*p* = 0.0862); however, the 3-year DFS rates were very good in both arms (78.2% in the XP/XRT/XP arm and 74.2% in the XP arm) when compared to the results of INT-0116. These favorable results might be in part explained from the high percentage of pts with early stage disease; approximately 60% of them in each arm had stage IB and II disease and therefore better prognosis. Besides the higher percentage of low risk group, another weakness of this study is the very small number of planned events, registered at the time of final analysis compared to the planned ones, which implies the need for an extended follow-up period, in order to get a clear view of results. Patient characteristics were similar between the two arms, with the exception of a slightly increased number of diffuse type tumors in the CRT arm (63% versus 57%), which have an unfavorable prognosis and more intestinal tumors (39% versus 33%) in the CT arm, but this parameter does not seem to interfere with the results. Interestingly, the subgroup of pts with LN involvement had superior DFS (*p* = 0.0365) when treated with CRT compared to CT, but this observation needs further validation. With the completion of 7 years of follow-up updated report was published. The effect of RT addition on DFS and OS differed by Lauren classification and lymph node involvement. Subgroup analyses also showed that CRT significantly improved DFS in node-positive disease and with intestinal-type GC [71].

Interestingly tolerability rates in the ARTIST trial were high throughout the study; treatment was completed as planned, in 75% in the XP arm and in 82% in the XP/XRT/XP arm. The rates of grades III and IV adverse events were low in both treatment groups. These results are impressive considering that in the INT-0116 trial the completion rate was only 63%. The younger median age in this trial (56 versus 62 years old in the INT-0116) and the favorable selection criteria may in part explain the toxicity and tolerability discrepancies.

Of special interest is the result of comparison of CRT use before and after 2001. Before 2001, postoperative CRT was used in 17% of the Oregon cases, while after 2001 use of CRT was doubled to 36.8% of cases. This increase was statistically significant (*p* < 0.001) but still almost two-thirds (63.2%) of pts did not receive adjuvant CRT, even though the results of INT116 were widely published and many other relative trials followed. Also in an updated analysis, they reported that 74% of the preoperatively treated pts completed therapy, compared with 34% of the postoperatively treated ones [42]. These notifications may suggest that clinicians are not yet persuaded for the results of CRT or they have second thoughts on tolerability and toxicity. The optimal regimen for postoperative CRT has not yet been established and the high relapse rate clearly indicates the need for improved systemic therapies. Since there are no phase III randomized data evaluating CRT versus adjuvant CT following less than D1 dissection adjuvant CRT remains one of the preferable strategies in this setting and particularly for them who have R1 resection. CRT strategy could also be considered in D1 resection as alternative of the perioperative approaches (MAGIC trial) for those referred after surgery, but its role for D2 resection has yet to be established.

Interesting and awaited ongoing trials that probably will refine our understanding in the area are shortly presented in Table 2. The ARTIST-II trial will study pts with involved LNs in the same design of the ARTIST trial evaluating CRT after a D2 resection. ARTIST-II is expected to give a better understanding of the optimal approach (adjuvant CT versus CRT) for the subgroup of pts with completely resected GC and nodal involvement. The CRITICS trial will evaluate the benefits of adding postoperative CRT to perioperative CT with S1/oxaliplatin and ECX, respectively [72]. Furthermore, the TOPGEAR trial will evaluate preoperative therapy options for gastric or GEJ adenocarcinomas comparing 3 cycles of preoperative ECF CT to 2 cycles of ECF followed by CRT prior to surgery.

Regarding targeted therapy, the AVAGAST trial failed to meet its endpoints and the ongoing MAGIC-B trial evaluating bevacizumab is awaited. Moreover, the ongoing TOXAG trial will study Trastuzumab with postoperative CRT and the RTOG 1010 will evaluate Trastuzumab in the perioperative or adjuvant setting. Additionally, due to the fact that the anti-EGFR antibodies (Cetuximab, Panitumumab) failed to improve outcomes in the metastatic disease in EXPAND and REAL3 trial, respectively, for the moment there is lack of evidence to be tested in the adjuvant setting [73, 74].

## 8. Conclusion

Until those trials are mature, one may question where do we stand more than 10 years after the INT-0116 since we practically walk around with the same original protocol. How do we evaluate all these discrepancies between the different trials? Do the small phase I-II trials with high diversity in design contributed to our knowledge or they just add some fog to the scenery? Probably before moving forward we should take a step back and redesign trials with special care to completeness and homogeneity in surgical staging, histological subtype, and use of a uniform language for response evaluation. Then we should reconsider the addition of other chemotherapy agents and further explore targeted agents role. Before extrapolating data to western populations, one should be mindful that the ACTS-GC, CLASSIC, and ARTIST trials were all conducted in East Asia. Studies are needed to duplicate the findings from Asia in Europe and USA, having in mind the differences in the biology and tumor characteristics.

Certainly, the optimal strategy remains to be defined. For the moment both adjuvant CRT and perioperative CT are acceptable options, while in Asia adjuvant CT is also used. Encouragement for the development of multicenter randomized trials will address the optimal sequence and timing of CT, RT in respect to surgery. Potential stratification factors like the level of nodal dissection, margin status, and potentially molecular profiling of the disease have to be further validated.

Doubtless, the one size fits all approach has failed and the combined modality treatment is the best option for cure. Biomarkers and methods of identifying pts with GC who are more likely to benefit from therapy are currently awaited and needed. Recently, the Cancer Genome Atlas Research Network identified four molecular subtypes of GC and the viable targets examined could advance clinical research in the near future.

## Figures and Tables

**Table 1:** Trials of curative strategy for gastric cancer.

Trial	Patients number	Intervention	OS	DFS
INT-0116	A: 275 B: 281	A: Surgery B: Surgery→CRT	HR = 1.32; *p* = 0.004	HR = 1.51; *p* < 0.001

CALGB-80101	A: 272 B: 274	A: Surgery→CRT B: Surgery→ECF/CRT/ECF	HR = 1.03; *p* = 0.8	HR = 1; *p* = 0.99

ARTIST	A: 228 B: 230	A: Surgery→XP B: Surgery→XP/CRT/XP	NR	HR = 0.687; *p* = 0.047

MAGIC	A: 253 B: 250	A: Surgery B: ECF→Surgery→ECF	HR = 0.75; *p* = 0.009	HR = 0.66; *p* < 0.001

ACTS-GC	A: 530 B: 529	A: Surgery B: Surgery→S-1	HR = 0.68; *p* = 0.002	HR = 0.62; *p* < 0.001

CLASSIC	A: 515 B: 520	A: Surgery B: Surgery→CAPOX	HR = 0.72; *p* = 0.049	HR = 0.75; *p* < 0.001

SAMIT	A: 359 B: 364 C: 355 D: 355	A: Surgery→UFT B: Surgery→S-1 C: Surgery→Pac/UFT D: Surgery→Pac/S-1	NR	(i) C + D versus A + B, HR = 0.92; *p* = 0.273 (ii) A + C versus B + D, HR = 1.23; NR

CRT: chemoradiation.

ECF: epirubicin/cisplatin/5-fluorouracil.

NR: not reported.

XP: cisplatin/capecitabine.

CAPOX: oxaliplatin/capecitabine.

**Table 2:** Ongoing trials in the adjuvant therapy setting for gastric cancer.

Trial	Phase/patients sample	Tumor location	Intervention	Primary endpoint
ARTIST II (NCT00407186)	III 1,000	Stomach/GEJ	A: Surgery→S-1 + RT/S-1 + CDDP + RT B: S-1/S-1 + CDDP	DFS

CRITICS (NCT00407186)	III 788	Stomach/GEJ	A: ECC/EOC→Surgery→CC + RT B: ECC/EOC→Surgery→ECC/EOC	OS

TOPGEAR	II/III 752	Stomach/GEJ	A: FU/C + RT→Surgery→ECF/EOX B: ECF/EOX→Surgery→ECF/EOX	OS

MAGIC-B (NCT00450203)	II/III 1,100	Stomach/GEJ	A: ECX + BEV→Surgery→ECX + BEV B: ECX + BEV→Surgery→ECX + BEV	OS

JCOG (NCT00252161)	III 316	Stomach/GEJ	A: S-1 + CDDP→Surgery→S-1 B: Surgery→S-1	OS

RTOG-1010 (NCT01196390)	III 480	Middle-lower esophageal/GEJ	A: C-PAC + RT + H→Surgery→H B: C-PAC + RT→Surgery	DFS

NCT01711242	III 300	Stomach	A: Surgery→XELOX B: Surgery→XELOX/RT/XELOX	DFS

TOXAG (NCT01748773)	II 40	Stomach/GEJ	Surgery→XELOX + H + RT	Safety/tolerability

RT: radiation therapy, 45 Gy.

S-1: tegafur/gimeracil/oteracil.

CDDP: cisplatin.

ECC: epirubicin/cisplatin/capecitabine.

EOC: epirubicin/oxaliplatin/capecitabine.

FU: 5-fluorouracil.

ECF: epirubicin/cisplatin/5-fluorouracil.

ECX: epirubicin/cisplatin/capecitabine.

BEV: bevacizumab.

C: carboplatin.

PAC: paclitaxel.

H: trastuzumab.

XELOX: oxaliplatin-capecitabine.

## References

[1] Lau M., Le A., El-Serag H. B. (2006). Noncardia gastric adenocarcinoma remains an important and deadly cancer in the United States: secular trends in incidence and survival. *The American Journal of Gastroenterology*.

[2] Ferlay J., Bray F., Parkin D. M. (2001). *Gobocan 2000: Cancer Incidence and Mortality Worldwide*.

[3] American Cancer Society (2008). *Cancer Facts & Figures*.

[4] Cunningham D., Allum W. H., Stenning S. P. (2006). Perioperative chemotherapy versus surgery alone for resectable gastroesophageal cancer. *The New England Journal of Medicine*.

[5] Hundahl S. A., Phillips J. L., Menck H. R. (2000). The National Cancer Data Base report on poor survival of U.S. gastric carcinoma patients treated with gastrectomy: fifth edition American Joint Committee on Cancer staging, proximal disease, and the different diseas hypothesis. *Cancer*.

[6] Hermans J., Bonenkamp J. J., Boon M. C. (1993). Adjuvant therapy after curative resection for gastric cancer: meta-analysis of randomized trials. *Journal of Clinical Oncology*.

[7] Gunderson L. L., Donohue J. H., Burch P. A. (1995). *Clinical Oncology*.

[8] Earle C. C., Maroun J. A. (1999). Adjuvant chemotherapy after curative resection for gastric cancer in non-Asian patients: revisiting a meta-analysis of randomised trials. *European Journal of Cancer*.

[9] http://www.uptodate.com.

[10] Paoletti X., Oba K., Burzykowski T. (2010). Benefit of adjuvant chemotherapy for resectable gastric cancer: a meta-analysis. *Journal of the American Medical Association*.

[11] Bang Y.-J., Kim Y.-W., Yang H.-K. (2012). Adjuvant capecitabine and oxaliplatin for gastric cancer after D2 gastrectomy (CLASSIC): a phase 3 open-label, randomised controlled trial. *The Lancet*.

[12] Sasako M., Kinoshita T., Furukawa H. (2010). Five-year results of the randomized phase III trial comparing S-1 monotherapy versus surgery alone for stage II/III gastric cancer patients after curative D2 gastrectomy (ACTS-GC study). *Annals of Oncology*.

[13] Boige V., Pignon J., Saint-Aubert B. (2007). Final results of a randomized trial comparing preoperative 5-fluorouracil/cisplatin to surgery alone in adenocarcinoma of stomach and lower esophagus (ASLE): FNLCC ACCORD07-FFCD 9703 trial. *Journal of Clinical Oncology*.

[14] Schuhmacher C., Gretschel S., Lordick F. (2010). Neoadjuvant chemotherapy compared with surgery alone for locally advanced cancer of the stomach and cardia: European organisation for research and treatment of cancer randomized trial 40954. *Journal of Clinical Oncology*.

[15] Nio Y., Koike M., Omori H. (2004). A randomized consent design trial of neoadjuvant chemotherapy with tegafur plus uracil (UFT) for gastric cancer—a single institute study. *Anticancer Research*.

[16] Ychou M., Boige V., Pignon J.-P. (2011). Perioperative chemotherapy compared with surgery alone for resectable gastroesophageal adenocarcinoma: an FNCLCC and FFCD multicenter phase III trial. *Journal of Clinical Oncology*.

[17] Macdonald J. S., Smalley S. R., Benedetti J. (2001). Chemoradiotherapy after surgery compared with surgery alone for adenocarcinoma of the stomach or gastroesophageal junction. *The New England Journal of Medicine*.

[18] Bunt A. M. G., Hermans J., Smit V. T. H. B. M., Van De Velde C. J. H., Fleuren G. J., Bruijn J. A. (1995). Surgical/pathologic-stage migration confounds comparisons of gastric cancer survival rates between Japan and Western Countries. *Journal of Clinical Oncology*.

[19] Ohtsu A., Yoshida S., Saijo N. (2006). Disparities in gastric cancer chemotherapy between the east and west. *Journal of Clinical Oncology*.

[20] Macdonald J. S. (2011). Gastric cancer: Nagoya is not New York. *Journal of Clinical Oncology*.

[21] Yu C., Yu R., Zhu W., Song Y., Li T. (2012). Intensity-modulated radiotherapy combined with chemotherapy for the treatment of gastric cancer patients after standard D1/D2 surgery. *Journal of Cancer Research and Clinical Oncology*.

[22] Kim T. H., Park S. R., Ryu K. W. (2012). Phase 3 trial of postoperative chemotherapy alone versus chemoradiation therapy in stage III-IV gastric cancer treated with R0 gastrectomy and D2 lymph node dissection. *International Journal of Radiation Oncology Biology Physics*.

[23] Zhu W.-G., Xua D.-F., Pu J. (2012). A randomized, controlled, multicenter study comparing intensity-modulated radiotherapy plus concurrent chemotherapy with chemotherapy alone in gastric cancer patients with D2 resection. *Radiotherapy and Oncology*.

[24] Kwon H.-C., Kim M. C., Kim K. H. (2010). Adjuvant chemoradiation versus chemotherapy in completely resected advanced gastric cancer with D2 nodal dissection. *Asia-Pacific Journal of Clinical Oncology*.

[25] Lee J., Lim D. H., Kim S. (2012). Phase III trial comparing capecitabine plus cisplatin versus capecitabine plus cisplatin with concurrent capecitabine radiotherapy in completely resected gastric cancer with D2 lymph node dissection: the ARTIST trial. *Journal of Clinical Oncology*.

[26] Ohri N., Garg M. K., Aparo S. (2013). Who benefits from adjuvant radiation therapy for gastric cancer? A meta-analysis. *International Journal of Radiation Oncology Biology Physics*.

[27] Landry J., Tepper J. E., Wood W. C., Moulton E. O., Koerner F., Sullinger J. (1990). Patterns of failure following curative resection of gastric carcinoma. *International Journal of Radiation Oncology, Biology, Physics*.

[28] Gunderson L. L., Sosin H. (1982). Adenocarcinoma of the stomach: areas of failure in a re-operation series (second or symptomatic look) clinicopathologic correlation and implications for adjuvant therapy. *International Journal of Radiation Oncology Biology Physics*.

[29] Moertel C. G., Childs D. S., O'Fallon J. R., Holbrook M. A., Schutt A. J., Reitemeier R. J. (1984). Combined 5-fluorouracil and radiation therapy as a surgical adjuvant for poor prognosis gastric carcinoma. *Journal of Clinical Oncology*.

[30] Dent D. M., Werner I. D., Novis B., Cheverton P., Brice P. (1979). Prospective randomized trial of combined oncological therapy for gastric carcinoma. *Cancer*.

[31] Moertel C. G., Childs D. S., Reitemeier R. J., Colby M. Y., Holbrook M. A. (1969). Combined 5-fluorouracil and supervoltage radiation therapy of locally unresectable gastrointestinal cancer. *The Lancet*.

[32] Schein P. S., Stablein D. M., Bruckner H. W. (1982). A comparison of combination chemotherapy and combined modality therapy for locally advanced gastric carcinoma. *Cancer*.

[33] Ng K., Meyerhardt J. A., Fuchs C. S. (2007). Adjuvant and neoadjuvant approaches in gastric cancer. *Cancer Journal*.

[34] Ajani J. A., Mansfield P. F., Janjan N. (2004). Multi-institutional trial of preoperative chemoradiotherapy in patients with potentially resectable gastric carcinoma. *Journal of Clinical Oncology*.

[35] Ajani J. A., Winter K., Okawara G. S. (2006). Phase II trial of preoperative chemoradiation in patients with localized gastric adenocarcinoma (RTOG 9904): quality of combined modality therapy and pathologic response. *Journal of Clinical Oncology*.

[36] Smalley S. R., Benedetti J. K., Haller D. G. (2012). Updated analysis of SWOG-directed intergroup study 0116: a phase III trial of adjuvant radiochemotherapy versus observation after curative gastric cancer resection. *Journal of Clinical Oncology*.

[37] Papadimitriou K., Vassiliou V., Kountourakis P., Polyviou P., Andreopoulos D., Papamichael D. (2012). Adjuvant chemoradiotherapy (CRT) for high-risk gastric cancer (GC) patients: single-center experience using infusional 5-fluorouracil (5FU) and radiotherapy (RT). *Medical Oncology*.

[38] Macdonald J. S., Benedetti J., Smalley S. R. (2009). Chemoradiation of resected gastric cancer: a 10-year follow-up of the phase III trial INT0116 (SWOG 9008). *Journal of Clinical Oncology*.

[39] Kim S., Lim D. H., Lee J. (2005). An observational study suggesting clinical benefit for adjuvant postoperative chemoradiation in a population of over 500 cases after gastric resection with D2 nodal dissection for adenocarcinoma of the stomach. *International Journal of Radiation Oncology, Biology, Physics*.

[40] Fiorica F., Cartei F., Enea M. (2007). The impact of radiotherapy on survival in resectable gastric carcinoma: a meta-analysis of literature data. *Cancer Treatment Reviews*.

[41] Valentini V., Cellini F., Minsky B. D. (2009). Survival after radiotherapy in gastric cancer: systematic review and meta-analysis. *Radiotherapy and Oncology*.

[42] Dikken J. L., Jansen E. P. M., Cats A. (2010). Impact of the extent of surgery and postoperative chemoradiotherapy on recurrence patterns in gastric cancer. *Journal of Clinical Oncology*.

[43] Enestvedt C. K., Diggs B. S., Shipley D. K., Thomas C. R., Billingsley K. G. (2009). A population-based analysis of surgical and adjuvant therapy for resected gastric cancer: are patients receiving appropriate treatment following publication of the intergroup 0116 results?. *Gastrointestinal Cancer Research*.

[44] Smalley S. R., Gunderson L., Tepper J. (2002). Gastric surgical adjuvant radiotherapy consensus report: rationale and treatment implementation. *International Journal of Radiation Oncology Biology Physics*.

[45] D'Angelica M., Gonen M., Brennan M. F., Turnbull A. D., Bains M., Karpeh M. S. (2004). Patterns of initial recurrence in completely resected gastric adenocarcinoma. *Annals of Surgery*.

[46] Bozzetti F., Marubini E., Bonfanti G., Miceli R., Piano C., Gennari L. (1999). Subtotal versus total gastrectomy for gastric cancer: five-year survival rates in a multicenter randomized Italian trial. Italian Gastrointestinal Tumor Study Group. *Annals of Surgery*.

[47] Bajetta E., Buzzoni R., Mariani L. (2002). Adjuvant chemotherapy in gastric cancer: 5-year results of a randomised study by the Italian trials in medical oncology (ITMO) group. *Annals of Oncology*.

[48] Gunderson L. L., Callister M. D., Jaroszewski D. E. (2009). Localized gastric or gastroesophageal cancer—chemoradiation is a pertinent component of adjuvant treatment for patients at high risk of relapse. *Gastrointestinal Cancer Research*.

[49] Bonenkamp J. J., Songun I., Hermans J. (1995). Randomised comparison of morbidity after D1 and D2 dissection for gastric cancer in 996 Dutch patients. *The Lancet*.

[50] Sasako M., Maruyama K., Kinoshita T., Bonenkamp J. J., van de Velde C. J., Hermans J. (1992). Quality control of surgical technique in a multicenter, prospective, randomized, controlled study on the surgical treatment of gastric cancer. *Japanese Journal of Clinical Oncology*.

[51] Robertson C. S., Chung S. C. S., Woods S. D. S. (1994). A prospective randomized trial comparing R1 subtotal gastrectomy with R3 total gastrectomy for antral cancer. *Annals of Surgery*.

[52] Cuschieri A., Fayers P., Fielding J. (1996). Postoperative morbidity and mortality after D1 and D2 resections for gastric cancer: preliminary results of the MRC randomised controlled surgical trial. *The Lancet*.

[53] Bonenkamp J. J., Hermans J., Sasako M., van de Velde C. J. H. (1999). Extended lymph-node dissection for gastric cancer. *The New England Journal of Medicine*.

[54] Sasako M. (1997). Risk factors for surgical treatment in the Dutch gastric cancer trial. *British Journal of Surgery*.

[55] Cuschieri A., Weeden S., Fielding J. (1999). Patient survival after D1 and D2 resections for gastric cancer: long-term results of the MRC randomized surgical trial. Surgical Co-operative Group. *British Journal of Cancer*.

[56] Songun I., Putter H., Kranenbarg E. M.-K., Sasako M., van de Velde C. J. (2010). Surgical treatment of gastric cancer: 15-year follow-up results of the randomised nationwide Dutch D1/D2 trial. *The Lancet Oncology*.

[57] Bunt A. M. G., Hogendoorn P. C. W., van de Velde C. J. H., Bruijn J. A., Hermans J. (1995). Lymph node staging standards in gastric cancer. *Journal of Clinical Oncology*.

[58] Schwartz G. K., Winter K., Minsky B. D. (2009). Randomized phase II trial evaluating two paclitaxel and cisplatin-containing chemoradiation regimens as adjuvant therapy in resected gastric cancer (RTOG-0114). *Journal of Clinical Oncology*.

[59] Kollmannsberger C., Budach W., Stahl M. (2005). Adjuvant chemoradiation using 5-fluorouracil/folinic acid/cisplatin with or without paclitaxel and radiation in patients with completely resected high-risk gastric cancer: two cooperative phase II studies of the AIO/ARO/ACO. *Annals of Oncology*.

[60] Kwon H. C., Kim M. C., Kim K. H. (2010). Adjuvant chemoradiation versus chemotherapy in completely resected advanced gastric cancer with D2 nodal dissection. *Asia-Pacific Journal of Clinical Oncology*.

[61] Kassam Z., Mackay H., Buckley C. A. (2010). Adjuvant chemoradiation for gastric cancer with infusional 5-fluorouracil and cisplatin: a phase I study. *Current Oncology*.

[62] Bouché O., Ychou M., Burtin P. (2005). Adjuvant chemotherapy with 5-fluorouracil and cisplatin compared with surgery alone for gastric cancer: 7-year results of the FFCD randomized phase III trial (8801). *Annals of Oncology*.

[63] Vaishampayan U. N., Ben-Josef E., Philip P. A. (2002). A single-institution experience with concurrent capecitabine and radiation therapy in gastrointestinal malignancies. *International Journal of Radiation Oncology Biology Physics*.

[64] Das P., Wolff R. A., Abbruzzese J. L. (2006). Concurrent capecitabine and upper abdominal radiation therapy is well tolerated. *Radiation Oncology*.

[65] Fuchs C. S., Tepper J. E., Niedzwiecki D. (2011). Postoperative adjuvant chemoradiation for gastric or gastroesophageal junction (GEJ) adenocarcinoma using epirubicin, cisplatin, and infusional (CI) 5-FU (ECF) before and after CI 5-FU and radiotherapy (CRT) compared with bolus 5-FU/LV before and after CRT: intergroup trial CALGB 80101. *Journal of Clinical Oncology*.

[66] Stahl M., Walz M. K., Stuschke M. (2009). Phase III comparison of preoperative chemotherapy compared with chemoradiotherapy in patients with locally advanced adenocarcinoma of the esophagogastric junction. *Journal of Clinical Oncology*.

[67] Fields R. C., Strong V. E., Gönen M. (2011). Recurrence and survival after pathologic complete response to preoperative therapy followed by surgery for gastric or gastrooesophageal adenocarcinoma. *British Journal of Cancer*.

[68] Díaz-González J. A., Rodríguez J., Hernández-Lizoain J. L. (2011). Patterns of response after preoperative treatment in gastric cancer. *International Journal of Radiation Oncology Biology Physics*.

[69] Pepek J. M., Chino J. P., Willett C. G. (2013). Preoperative chemoradiotherapy for locally advanced gastric cancer. *Radiation Oncology*.

[70] Lee J., Lim D. H., Kim S. (2014). Phase III trial to compare capecitabine/cisplatin (XP) versus XP plus concurrent capecitabine-radiotherapy in gastric cancer (GC): the final report on the ARTIST trial. *Journal of Clinical Oncology*.

[71] Park S. H., Sohn T. S., Lee J. (2015). Phase III trial to compare adjuvant chemotherapy with capecitabine and cisplatin versus concurrent chemoradiotherapy in gastric cancer: final report of the adjuvant chemoradiotherapy in stomach tumors trial, including survival and subset analyses. *Journal of Clinical Oncology*.

[72] http://clinicaltrials.gov.

[73] Lordick F., Kang Y.-K., Chung H.-C. (2013). Capecitabine and cisplatin with or without cetuximab for patients with previously untreated advanced gastric cancer (EXPAND): a randomised, open-label phase 3 trial. *The Lancet Oncology*.

[74] Waddell T., Chau I., Cunningham D. (2013). Epirubicin, oxaliplatin, and capecitabine with or without panitumumab for patients with previously untreated advanced oesophagogastric cancer (REAL3): a randomised, open-label phase 3 trial. *The Lancet Oncology*.

